# The continuing arc toward phototropic enlightenment

**DOI:** 10.1093/jxb/eraa005

**Published:** 2020-01-07

**Authors:** Emmanuel Liscum, Patrick Nittler, Katelynn Koskie

**Affiliations:** 1 C.S. Bond Life Sciences Center, University of Missouri, Columbia, MO, USA; 2 Division of Biological Sciences, University of Missouri, Columbia, MO, USA; 3 University of Missouri, USA

**Keywords:** NPH3, phosphorylation, phot1, phot2, phototropin, phototropism

## Abstract

Phototropism represents a simple physiological mechanism—differential growth across the growing organ of a plant—to respond to gradients of light and maximize photosynthetic light capture (in aerial tissues) and water/nutrient acquisition (in roots). The phototropin blue light receptors, phot1 and phot2, have been identified as the essential sensors for phototropism. Additionally, several downstream signal/response components have been identified, including the phot-interacting proteins NON-PHOTOTROPIC HYPOCOTYL 3 (NPH3) and PHYTOCHROME SUBSTRATE 4 (PKS4). While the structural and photochemical properties of the phots are quite well understood, much less is known about how the phots signal through downstream regulators. Recent advances have, however, provided some intriguing clues. It appears that inactive receptor phot1 is found dispersed in a monomeric form at the plasma membrane in darkness. Upon light absorption dimerizes and clusters in sterol-rich microdomains where it is signal active. Additional studies showed that the phot-regulated phosphorylation status of both NPH3 and PKS4 is linked to phototropic responsiveness. While PKS4 can function as both a positive (in low light) and a negative (in high light) regulator of phototropism, NPH3 appears to function solely as a key positive regulator. Ultimately, it is the subcellular localization of NPH3 that appears crucial, an aspect regulated by its phosphorylation status. While phot1 activation promotes dephosphorylation of NPH3 and its movement from the plasma membrane to cytoplasmic foci, phot2 appears to modulate relocalization back to the plasma membrane. Together these findings are beginning to illuminate the complex biochemical and cellular events, involved in adaptively modifying phototropic responsiveness under a wide varying range of light conditions.

## Teaser

Recent advances in the biochemical and cellular mechanisms of phototropin-dependent signaling are discussed.

## Introduction

**Fig. 1.**
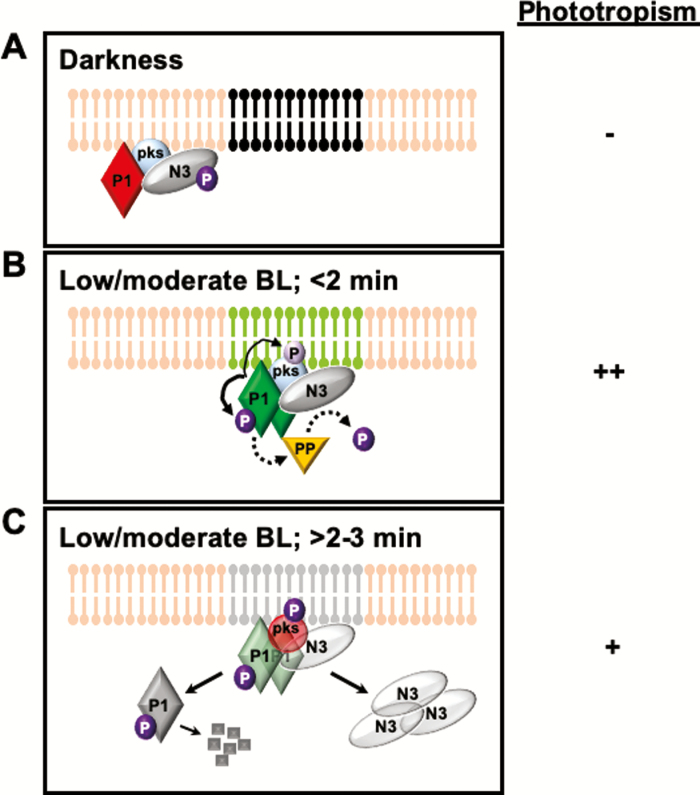
Model for phot1-associated early events in phototropic responsiveness under low to moderate light in etiolated seedlings. The plasma membrane lipid bilayer is shown in pink, black, light green, and gray; where the black and green areas designate sterol-rich microdomains that are phot inactive and active, respectively. Gray areas of the membrane are presumed to represent phot-active regions. Solid arrows represent known and characterized events, whereas dashed lines represent experimentally inferred events without a characterized mechanism. The relative phototropic responsiveness is given to the right for each of the light conditions: –, no or very little response; +, weak response; ++, moderate to strong response. (A) The basal phot1 signaling complex in etiolated seedlings kept in darkness. The complex is found dispersed on the inner face of the plasma membrane outside of sterol-rich microdomains. Phot1 (P1) is inactive (solid red); PKS4 (pks) is unphosphorylated and inactive; NPH3 (N3; solid gray) is fully phosphorylated (deep purple P) and presumed inactive. (B) The phot1 complex in etiolated seedlings exposed to low or moderate BL for a short duration (<2 min). Phot1 is activated (green) and moves into clusters within sterol-rich regions of the membrane (presumably with its interacting proteins), where it dimerizes and *trans*-autophosphorylates (purple P) and phosphorylates PKS4. Low levels of PKS4 phosphorylation (light purple P) are associated with positive regulation of phototropism. Phot1 activation also leads to the activation of an as yet identified protein phosphatase (PP) that dephosphorylates NPH3. (C) The phot1 complex in etiolated seedlings that received low or moderate BL continuously for a moderate time (>2–3 min). While some phot1 is retained in an active state (transparent green) at the plasma membrane, and can continue to phosphorylate PKS4 (represented by the deep purple P), some phot1 (gray, and presumed inactive) is translocated to the cytoplasm. Some of the cytoplasmic phot1 is degraded via a 26S proteasome-dependent process (Roberts *et al.*, 2011). Enhanced phosphorylation of PKS4 results in the conversion of this molecule to a repressor of phot1-dependent phototropism (pks in red with attached deep purple P). Dephosphorylated NPH3 (transparent gray) translocates from the plasma membrane to cytoplasmic aggregates. Each of these events results in down-regulation of phot1 signaling and response desensitization. While the phot1 complex is depicted as being localized to a sterol-rich region of the plasma membrane in (C), this is color-coded gray because it has not been experimentally determined.

Lacking locomotion to seek out food and safety, plants instead rely on a variety of responses that allow them to fulfill these needs and adapt to changes in their environment (Huang, 2006; Mizutani and Kanaoka, 2018). Responses to alterations in the local light environment include phototropism, changes in leaf orientation, and chloroplast movements (de Witt *et al*., 2016; Fiorucci and Fankhauser, 2017). Each of the aforementioned responses requires perception of blue light (BL) by a class of photoreceptors known as the phototropins (phots) (Galvao and Fankhauser, 2015; de Witt *et al*., 2016). Though gene copy number can vary depending upon plant species, there are two phot isoforms, phot1 and phot2; both associate primarily with the plasma membrane (Fig. 1A), and can exhibit shared and unique functions (Morrow *et al.*, 2018). Bimodular in structure, both phots contain a pair of LOV (light, oxygen, and voltage) domains that form an N-terminal sensory region, and a protein kinase domain (PKD) of the AGCVII subfamily of protein kinases as a C-terminal output domain (Morrow *et al.*, 2018). One of the key molecules involved in the transduction of phot signals is NON-PHOTOTROPIC HYPOCOTYL 3 (NPH3) (Liscum and Briggs, 1995, 1996; Motchoulski and Liscum, 1999; Inada *et al.*, 2004; Inoue *et al.*, 2008*a*; de Carbonnel *et al.*, 2010; Harada *et al.*, 2013), a founding member of the plant-specific NRL (NPH3/RPT2-Like) family of proteins (Pedmale *et al.*, 2010; Liscum *et al.*, 2014; Christie *et al.*, 2018). NPH3 and NRL family members are generally trimodular in structure (Pedmale *et al.*, 2010), with an N-terminal BTB (broad complex, tramtrack, and bric-a-brac) protein–protein interaction domain (Stogios *et al.*, 2005), a central ‘NPH3 domain’ (PFam, PF0300) of conserved amino acid sequence across the NRL family, and a C-terminal coiled-coil (CC) protein–protein interaction domain (Motchoulski and Liscum, 1999). The CC and BTB domains of NPH3 have been shown to mediate direct interaction with phot1 (Fig. 1A) and CULLIN3 (CUL3), respectively (Motchoulski and Liscum, 1999; Roberts *et al.*, 2011). These protein interactions facilitate ubiquitination (both mono- and polyubiquitination) of phot1 by a CULLIN3-Ring E3 ubiquitin ligase complex, CRL3^NPH3^, in which NPH3 functions as a substrate adaptor (Roberts *et al.*, 2011). The phot signaling capacity of NPH3 appears to be linked to its phosphorylation status, which is itself modulated by light condition and phot activity (Motchoulski and Liscum, 1999; Pedmale and Liscum, 2007;Tsuchita-Mayama *et al*., 2008; Haga *et al.*, 2015). Here we will discuss recent studies (see Box 1) that are beginning to address some of the most pressing questions, such as: what proteins are substrates for the phot PKD and how do these proteins influence phot signaling; how do post-translational modifications of phots and NPH3 alter signaling; and how does light-dependent re-localization of NPH3 influence signaling?

## Regulation of phot signaling via phot-dependent phosphorylation

It has been well established that BL-activated *trans*-autophosphorylation of phots (Fig. 1B) is critical for signaling function (Inoue *et al*., 2008*b*, 2011; Tseng and Briggs, 2010; Peterson *et al.*, 2017). Yet much less is known about the downstream targets of phots (Christie *et al.*, 2011; Demarsy *et al.*, 2012; Takemiya *et al.*, 2013; Hiyama *et al.*, 2017), or how phosphorylation of such proteins regulates phot-dependent processes. One such target protein is PHYTOCHROME KINASE SUBSTRATE 4 (PKS4). It was previously determined that PKS4 acts as a positive regulator of phototropism under low light conditions (Lariguet *et al.*, 2006; Demarsy *et al.*, 2012; Kami *et al.*, 2014; Fig. 1B), though phosphorylation of PKS4 is not essential for phototropic responsiveness (Demarsy *et al.*, 2012). Interestingly, it was found that PKS4 phosphorylation may play a role in a negative feedback control, though the mechanism remained unknown (Demasry *et al*., 2012). Schumacher *et al*. (2018) have now identified a potential mechanism (see Box 1).

**Fig. 2.**
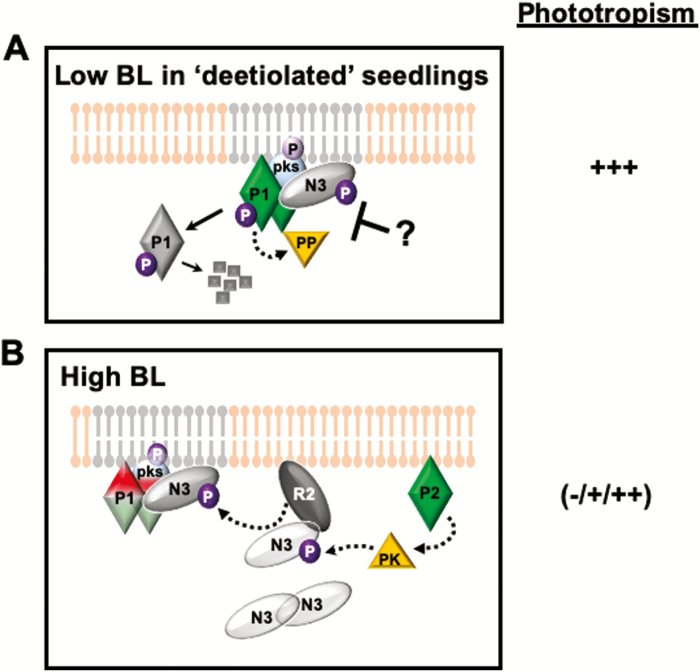
Model for phot1-associated early events in phototropic responsiveness in de-etiolated seedlings (A) or etiolated seedlings exposed to high intensity light for extended periods. The plasma membrane lipid bilayer is shown in pink and gray; where the gray areas designate presumed sterol-rich microdomains that are phot active. Solid arrows represent known and characterized events, whereas dashed lines represent experimentally inferred events without a characterized mechanism. The relative phototropic responsiveness is given to the right for each of the light conditions: –, no or very little response; +, weak response; +++, strong enhanced response. (A) The phot1 complex in de-etiolated seedlings exposed to low to moderate BL. De-etiolation results in the persistence of phosphophorylated NPH3 and its retention at the plasma membrane. Though the mechanism(s) for this is currently unknown, it would appear to influence either the protein phosphatase that dephosphorylates NPH3 in response to light, or the protein kinase that phosphorylates it. Phot1 is shown as an active (green) dimer associated with a presumed sterol-rich plasma membrane microdomain, and as a presumed inactive (green) cytoplasmic monomer which can be degraded by a 26S proteasome (Roberts *et al.*, 2011). PKS4 is presumed to be in its active form as the seedlings are highly phototropic. (B) The phot1 complex in etiolated seedlings exposed to high BL. Short-term exposure to high BL results in PKS4-dependent (red pks; deep purple P) suppression of phot1 (red) activity and thus weak phototropism. Exposure to high BL for extended periods (>30 min) results in the expression and activity of phot2 (P2) and RPT2 (R2), both of which stimulate the relocalization of NPH3 from the cytoplasm to the plasma membrane. Though the mechanism by which RPT2 does this is unknown, NPH3 relocalization does require phosphorylation (deep purple P) by an unknown protein kinase (PK) that appears to be activated in response to phot2 activity. Because phototropism is stronger in extended versus short-term high BL, but similar to that observed in etiolated seedlings exposed to low/moderate BL for short periods, it is presumed that both PKS4 (light blue pks; light purple P) and phot1 (light green) activity are moderate. Phot2 and RPT2 relocalization of NPH3 in etiolated plants results in resensitization of phot1 signaling that allows for adaptive responses in high light conditions.

By mutating the critical residue (Ser299) in PKS4 that is phosphorylated by phot1, to either prevent phosphorylation (PKS4-S299A) or generate a constitutive phosphorylation mimic (PKS4-S299D), Schumacher *et al*. (2018) found that the phosphorylation status of PKS4 acts as a ‘rheostat’ to mediate phototropic responsiveness under varying light intensities (Figs 1 and 2). Specifically, they found that under low light conditions, the PKS4-S299A mutant lines displayed phototropic curvatures that were temporally and quantitatively similar to those of the wild type and transgenic *pks4* mutants expressing a PKS4-WT protein (Schumacher *et al.*, 2018). However, in comparison with the response under low light, phototropism is both delayed and repressed under high light conditions in the wild type and *pks4* PKS4-WT transgenics, but neither of these alterations is observed in the PKS4-S299A mutants, suggesting that phosphorylated PKS4 may act as an inhibitor of the phototropism (Schumacher *et al.*, 2018). Consistent with this conclusion, the authors found that PKS4-S299D mutants exhibited slower and dampened phototropic responses under low light conditions, much like those observed in wild-type plants exposed to high light (Schumacher *et al.*, 2018). Thus, as the light intensity increases, so does the amount of phosphorylated PKS4, resulting in a progressively slower and weaker phototropic response. Schumacher *et al*. (2018) hypothesize that this sequence of events allows the plant to adapt appropriately to ever-changing light conditions in the natural diurnal environment. For instance, when a plant experiences a gradient of directional light but is in non-photosynthetically limiting light conditions, there would be no advantage to expending energy to exhibit a phototropic response. By examining the phototropic responses under conditions where plants are exposed to the same relative directional gradient of light but given at different light intensities, it was indeed found that wild-type seedlings exhibited ever decreasing phototropic curvature with increasing intensity (Schumacker *et al*., 2018). Strikingly, this response is dramatically diminished in the PKS4-S299A mutant (Schumacker *et al*., 2018).

Despite advances like that just discussed, the dearth of identified phot kinase substrates severely limits our understanding of phot-dependent signal transduction. To date, just five substrates have been reported: the phots themselves (Deng *et al.*, 2014; Inoue *et al.*, 2008*b*, 2011; Sullivan *et al.*, 2008); PKS4 (Demarsy *et al.*, 2012); BLUS1 (Takemiya *et al.*, 2013); and CBC1 (Hiyama *et al.*, 2017). In a recent report, Schnabel *et al*. (2018) described the development of a method to identify potential phot kinase substrates via thiophosphorylation (see Box 1). In brief, the authors generated mutant ‘gatekeeper’ (Emrick *et al.*, 2006; Allen *et al.*, 2007) versions of the phots by substituting a glycine for a conserved threonine within the β5 strand in the N lobe of the kinase domain (Kornev and Taylor, 2015), thus allowing the accommodation of a bulky ATP analog, *N*^6^-benzyl-ATPγS (Schnabel *et al.*, 2018). The phot gatekeeper mutants, called Cerberus for the ‘keeper’ of the underworld in Greek mythology, were shown to undergo *trans*-auto-thiophosphorylation *in vitro* (Schnabel *et al.*, 2018). Moreover, both phot1- and phot2-Cerberus were found to thiophosphorylate BLUS1 (Schnabel *et al.*, 2018), a known phot1 target (Takemiya *et al.*, 2013). Interestingly, phot1-Cerberus expressed transgenically in Arabidopsis could still utilize native ATP and was capable of functionally complementing both loss of phototropism and leaf flattening in a *phot1phot2* double mutant (Schnabel *et al.*, 2018). This chemical genetic approach therefore represents a powerful tool to identify potential direct substrates of phot kinases, a potential game-changer indeed.

## Activation of phot1 in membrane microdomains

In darkness, phots associate with the cytoplasmic face of the plasma membrane (Fig. 1A) via the C-terminal portion of the PKD through an as yet unknown mechanism (Kong *et al*., 2007, 2013*a*, *b*). Upon BL absorption, phots are autophosphorylated (Fig. 1B), probably *in trans* through BL-induced dimerization of the receptor (Kaiserli *et al.*, 2009; Sullivan *et al.*, 2010; Petersen *et al*., 2017). With increasing exposure time, some portion of phot is internalized to the cytoplasm (Sakamoto and Briggs, 2002; Kong *et al.*, 2006; Han *et al.*, 2008; Wan *et al.*, 2008; Kaiserli *et al.*, 2009; Sullivan *et al.*, 2010; Fig. 1C). Although Preuten *et al*. (2015) have shown that internalization of phot1 is not necessary for primary signaling, it had also been reported that phot1 exhibits mosaic patterns and forms punctate aggregates at the surface of the membrane in response to BL exposure (Liscum, 2016), leaving open the question of whether dynamic BL-induced phot movements at the membrane might be involved in signal propagation.

Xue *et al*. (2018) have provided the first steps toward addressing this aforementioned question by utilizing single particle imaging (FRET-FLIM, Li *et al.*, 2013; Wang *et al.*, 2015; VA-TIRFM, Wan *et al.*, 2011) to assess the dynamic movement of phot1–green fluorescent protein (GFP) within the plasma membrane (see Box 1). The authors found that in darkness phot1 is broadly distributed at the inner surface of the membrane as monomeric units (Fig. 1A). Upon BL exposure, phot1 dimerizes and aggregates into clusters on the inner surface of the plasma membrane (Xue *et al.*, 2018; Fig. 1B), consistent with previous observation by spinning-disc confocal microscopy (Liscum, 2016). Moreover, it was found that phot1–GFP co-localizes with AtRem1.3, a marker for sterol-rich lipid environments (Demir *et al.*, 2013), indicating that these phot1-containing foci are found within sterol-rich microdomains (Xue *et al.*, 2018; Fig. 1B). Interestingly, while BL-induced dimerization appears to be essential for movement of phot1 to the sterol-rich microdomains, *trans*-autophosphorylation is not (Xue *et al.*, 2018). Rather, *trans*-autophosphorylation appears to occur within the membrane microdomains containing dimeric phot1 (Xue *et al.*, 2018; Fig. 1B). Findings that sterol depletion compromises phototropic responsiveness (Xue *et al.*, 2018), together with previous results indicating that phot1 autophosphorylation is prerequisite for phototropism (Inoue *et al.*, 2008*b*), strongly suggest that phot1 signaling occurs from these sterol-rich foci (Fig. 1B).

## An NPH3 rheostat: phosphorylation status and localization regulate phot1-dependent phototropism

As discussed earlier, NPH3 is a phot1-interacting protein that is part of a CRL3^NPH3^ ubiquitin ligase complex necessary for phototropism (Liscum and Briggs, 1995, 1996; Motchoulski and Liscum, 1999; Inada *et al.*, 2004; Roberts *et al.*, 2011). Though reversible phosphorylation of NPH3 has been shown to be important for phototropic responsiveness (Motchoulski and Liscum, 1999; Pedmale and Liscum, 2007; Tsuchita-Mayama *et al*., 2008; Haga *et al.*, 2015), the mechanistic impact of this post-translational modification has remained unknown. A recent study has, however, begun to shed some light on this open question (Sullivan *et al.*, 2019; see Box 1).

Previous studies have shown that phototropism is enhanced in de-etiolated as compared with etiolated seedlings of several species (Hart and Macdonald, 1981; Gordon *et al.*, 1982; Ellis, 1987), including Arabidopsis (Jin *et al.*, 2001). Sullivan *et al*. (2019) found that a reduction in dephosphorylation of NPH3, as well as its retention at the plasma membrane, underpins this phytochrome- and cryptochrome-dependent enhancement of phototropism (Liscum *et al.*, 2014) in de-etiolated Arabidopsis seedlings. In etiolated (or dark-adapted) seedlings, NPH3 exists predominantly in a phosphorylated state, and associates with the plasma membrane (Motchoulski and Liscum, 1999; Haga *et al.*, 2015; Fig. 1A). In response to BL exposure, the dephosphorylated isoform accumulates (Motchoulski and Liscum, 1999; Pedmale and Liscum, 2007; Fig. 1B) and moves from the plasma membrane to form cytoplasmic aggregates in a phot1-dependent fashion (Haga *et al.*, 2015; Fig. 1C). Surprisingly, given these previous findings, Sullivan *et al*. (2019) found that the phosphorylated form of NPH3 also predominates in de-etiolated seedlings. More surprising still, unlike what occurs in etiolated seedlings, much less NPH3 is dephosphorylated in de-etiolated seedlings exposed to unilateral BL (Sullivan *et al.*, 2019). Interestingly, the higher levels of phosphorylated NPH3 in de-etiolated as compared with etiolated seedlings exposed to low to moderate intensity phototropic stimuli (Fig. 1B) also resulted in higher retention of NPH3 at the plasma membrane (Sullivan *et al.*, 2019) where it interacts with phot1 (Lariguet *et al*., 2006; Roberts *et al.*, 2011; Haga *et al.*, 2015). Based on these observations, Sullivan *et al*. (2019) hypothesize that it is the proportion of phosphorylated NPH3 interacting with phot1 at the plasma membrane that determines the phototropic responsiveness.

## NPH3 as a regulator of high light-induced, phot2-dependent phototropism

Under conditions where phot1 function is redundant to phot2, such as during the induction of phototropism by high intensity BL (Sakai *et al.*, 2001; Inada *et al.*, 2004), it can be difficult to isolate the phot2-dependent properties of signaling from those of phot1. Zhao *et al*. (2018) recently tackled this problem by screening for mutants in a *phot1*-null background that had impaired phot2-dependent phototropism under high BL. Perhaps not surprisingly, given its apparent essential role for phototropic signaling under a wide range of light intensities (Liscum *et al.*, 2014; Fankhauser and Christie, 2015), one of these mutants turned out to be a new allele of *NPH3* (Zhao *et al.*, 2018; see Box 1). The surprises came when NPH3 localization was examined under high BL in various genetic backgrounds (wild type, *phot1*, *phot2*, *phot1phot2*, *rpt2*, or *pks1pks2pks4*; Zhao *et al.*, 2018). First, as observed previously for etiolated seedlings exposed to low BL (Haga *et al.*, 2015), NPH3 was found in clusters at the plasma membrane in darkness independent of genotype (Fig. 1A), and was relocalized into cytoplasmic aggregates in wild-type seedlings in a phot1-dependent fashion in response to high BL exposure (Zhao *et al.*, 2018; Fig. 2B). However, high BL-induced cytoplasmic aggregation of NPH3 was found to be enhanced in the *phot2* background (Zhao *et al.*, 2018). Secondly, again similarly to what occurs in low BL (Haga *et al.*, 2015), prolonged irradiation with high BL results in the RPT2-dependent relocation of cytoplasmic NPH3 back to the plasma membrane in etiolated wild-type seedlings, but this relocalization did not occur in the *phot2* or *rpt2* backgrounds (Zhao *et al.*, 2018). Together these findings indicate that phot1 regulates the dissociation of NPH3 from the plasma membrane into cytoplasmic aggregates in high BL as a means of sensory response desensitization (Zhao *et al.*, 2018), as occurs in low BL (Haga *et al.*, 2015) (see Fig. 1C); whereas, both phot2 and RPT2 regulate the relocation of NPH3 from cytosol to the plasma membrane, and thus ‘reconstruction’ of a phototropically active phot1–NPH3 complex, as a means to acclimate to prolonged high BL exposure (Zhao *et al.*, 2018) (see Fig. 2B).

## Concluding remarks and future directions

While the recent findings highlighted here are both exciting and enlightening, like any good science they beg more questions. For example, how do phosphorylated isoforms of PKS4 inhibit phot1-dependent phototropism? Is it through a direct interaction with, and modulation of, phot1? Or does it require other yet identified components? Given that PKS proteins are intrinsically disordered proteins (Schumacker *et al*., 2018), answers to these questions may provide insight into the function of other disordered proteins (Wright and Dyson, 2015) in phot-dependent signaling, such as NPH3 (Sullivan *et al.*, 2019). How does phot1 partitioning to sterol-rich membrane microdomains in response to phototropic stimulation link phot signaling to alterations in auxin transport that ultimately drive differential growth (Morrow *et al.*, 2018; Zhang *et al.*, 2017)? Does PKS4 phosphorylation status influence phot1 partitioning in these microdomains? How does the ‘NPH3 phosphorylation rheostat’ impact this cell biology? What protein phosphatase(s) and protein kinase(s) regulate the phot1- and phot2-induced changes in NPH3 phosphorylation and localization? How does NPH3 phosphorylation state impact its function within the CRL3^NPH3^ ubiquitin ligase, and how does that alter phot1 ubiquitination and function? Also, how are the PKS4-dependent inhibition of phot1 function and phot2-dependent re-sensitization of phot1 signaling capacity integrated in natural environments to allow for enhanced phototropism in de-etiolated seedlings? These and many other questions remain, but, thanks to the studies discussed here, paths forward have been illuminated.

Box 1. **Key developments in understanding the phototropin-dependent signaling leading to phototropism**

- PKS4 phosphorylation status regulates phot1 signaling strength

Schumacker *et al*. (2018) showed that phot1-dependent phosphorylation of PKS4 leads to a negative feedback regulation of phot1-induced phototropism. Under low blue light (BL), PKS4 exists largely in an unphosphorylated state and functions as a positive regulator of phot1-dependent phototropism. As the BL intensity increases, phot1 directly phosphorylates more and more PKS4, promoting a shift in PKS4 action from positive to negative regulator. The greater the proportion of PKS4 phosphorylated, the more phototropism is diminished.

- A gatekeeper thiophosphorylation method to identify phototropin kinase substrates

Schnabel *et al.* (2018) have developed mutant phototropins, called phot-Cerberus, in which the PKD is mutated to allow *N*^6^-benzyl-APγãS to be utilized as an ATP source for subsequent thiophosphorylation of phot substrates. Both phot1- and phot2-Cerberus can thio-autophosphorylate in a BL-dependent fashion; and both can thiophosphorylate BLUS1, a known *in planta* substrate of the phototropins. Moreover, phot1-Cerberus can utilize endogenous ATP and functionally complement the aphototropic phenotype of a *phot1phot2* double mutant. This represents a powerful tool for substrate discovery, *in vitro* and *in vivo*.

- Sterol-rich plasma membrane microdomains as the site of phot1 activation

Xue *et al.* (2018) have utilized single particle FRET-FLIM/VA-TIRFM microscopic analysis to characterize the mono-/dimeric state, and intra-plasma membrane dynamics, of phot1 in response to BL exposure. It was found that phot1 exists predominantly as dispersed monomers at the inner surface of the plasma membrane in darkness, but rapidly dimerizes and forms aggregate clusters with sterol-rich microdomains. It appears that these clusters are the site of BL-induced *trans*-autophosphorylation, rather than the phosphorylation being a driver for intracellular movement. It is presumed that these sterol-rich phot1-containing microdomains are the sites of initial signal transduction/integration.

- An NPH3 phosphorylation and localization ‘rheostat’ that regulates phot1-dependent phototropism

Sullivan *et al.* (2019) demonstrated that enhanced phototropic responsiveness observed in de-etiolated seedlings results from retention of phosphorylated NPH3 at the plasma membrane. In etiolated seedlings, and dark-adapted de-etiolated seedlings, NPH3 is found predominantly in its phosphorylated form at the inner surface of the plasma membrane, where it interacts with phot1. In response to directional BL, NPH3 is rapidly dephosphorylated and moves to the cytoplasm where it forms aggregates. De-etiolation (the shift from heterotrophy to autotrophy) results in a much higher proportion of phosphorylated NPH3 being present and retained at the plasma membrane, even after exposure to directional BL, and is positively correlated with enhanced phototropic responsiveness in de-etiolated seedlings. Treatment of etiolated seedlings with the protein phosphatase inhibitor OKA also reduces BL-induced dephosphorylation of NPH3 and its movement to the cytoplasm, suggesting that the phosphorylation status of NPH3 is a key determinant of phot1-dependent phototropic responsiveness.

- High BL-induced phototropism requires phot2-dependent relocation of NPH3

Zhao *et al.* (2018) identified NPH3 as a crucial component of the phot2-regulated phototropism and demonstrated that phot2, like phot1, modulates the localization of NPH3. Unlike phot1, which promotes the dephosphorylation of NPH3 and its subsequent movement to cytoplasmic foci in response to BL, phot2 appears to modulate the relocalization of cytoplasmic NPH3 to the plasma membrane in response to high BL. RPT2, an NPH3 paralog, whose expression is light induced also appears to serve a similar function, though the mechanism is probably distinct. Differing regulation of the localization of NPH3, a critical phototropic signaling component, by the phototropins provides a dynamic means for adaptation and acclimation under varying light conditions.
